# DNA methylation entropy is a biomarker for aging

**DOI:** 10.18632/aging.206220

**Published:** 2025-03-12

**Authors:** Jonathan Chan, Liudmilla Rubbi, Matteo Pellegrini

**Affiliations:** 1 Computational and Systems Biology Interdepartmental Program at University of California, Los Angeles, Los Angeles, CA 90095, USA; 2 Department of Molecular, Cell and Developmental Biology, University of California, Los Angeles, Los Angeles, CA 90095, USA

**Keywords:** entropy, DNA methylation, aging, epigenetics, epigenetic clocks

## Abstract

The dynamic nature of epigenetic modifications has been leveraged to construct epigenetic clocks that accurately predict an individual’s age based on DNA methylation levels. Here we explore whether the accumulation of epimutations, which can be quantified by Shannon’s entropy, changes reproducibly with age. Using targeted bisulfite sequencing, we analyzed the associations between age, entropy, and methylation levels in human buccal swab samples. We find that epigenetic clocks based on the entropy of methylation states predict chronological age with similar accuracy as common approaches that are based on methylation levels of individual cytosines. Our approach suggests that across many genomic loci, methylation entropy changes reproducibly with age.

## INTRODUCTION

Somatic cells in the human body share the same genome, yet they must differentiate into diverse cell types to perform the vast array of tasks required to sustain life. This is made possible through epigenetic modifications, which include covalent changes to DNA that impact gene expression across cell types. The most widely studied epigenetic modification consists of the methylation of cytosine. DNA methyltransferases catalyze the conversion of cytosines to 5-methylcytosines (5mC), which preferentially occurs at CpG dinucleotides. The methylation of CpG islands (regions with high densities of CpG sites) in regulatory sequences can lead to the suppression of gene expression [1–3].

Previous studies have shown that DNA methylation is involved in X-chromosome inactivation [4–6] and the regulation of imprinted gene expression [7]. A growing body of evidence also points to a relationship between DNA methylation and aging. Methylation of many CpG islands is positively correlated with aging, while other loci not in CpG islands are negatively correlated with age [8]. Many bivalent chromatin domain promoters in blood have also been found to become hypermethylated with age [9]. These loci are associated with developmental genes that are commonly hypermethylated in cancers, pointing to a mechanistic link between aberrant hypermethylation, cancer, and aging. These age-related changes in DNA methylation have been extensively studied and are referred to as epigenetic drift [10]. This phenomenon has led researchers to build epigenetic clocks with the goal of predicting the age of an organism from its methylation profile. The blood-based epigenetic clock by Hannum et al. [11] and the multi-tissue clock by Horvath [12] yield age estimates with a correlation to chronological age well above r=0.90. DNA methylation data has also been used to produce epigenetic biomarkers that predict lifespan and age-related physiological changes [13–15].

Though aging is a complex phenomenon that remains to be fully understood, recent evidence demonstrates that cellular damage, which results in the loss of epigenetic information, plays a crucial role in senescence. Yang et al. developed a system called “ICE” (inducible changes to the epigenome) that accelerated the physiological and epigenetic aging process in mice via double-stranded DNA breaks without mutations [16]. The symptoms of aging were reversed via expression of a subset of Yamanka factors. Another study in mice found that CpG island methylation patterns were more ordered in stem cells of slow-proliferating tissues compared to fast-proliferating tissues, suggesting that DNA methylation events over time accumulate stochastically and can serve as a biomarker for aging [17]. However, it is not known whether the loss of epigenetic information can be used to predict chronological age in humans, and how such an approach might compare to traditional methods of epigenetic analysis.

Methylation microarrays have been widely used to measure the methylation levels of single CpGs [18, 19]. Another common approach is the use of bisulfite sequencing to measure the methylation of cytosine based on cytosine to thymine conversion rates [20, 21]. Bisulfite sequencing captures methylation states across CpGs of whole reads, allowing for methylation quantification methods that account for heterogeneity within profiled cell populations [22]. Xu et al. analyzed bisulfite sequence data to demonstrate that the Cellular Heterogeneity-Adjusted cLonal Methylation (CHALM), a measure of read level rather than single cytosine level methylation, provides better correlation with gene expression than the methylation of single cytosines [23]. However, neither the average methylation nor CHALM enable the calculation of methylation patterns across single DNA molecules.

To investigate age-related patterns in DNA methylation, we performed targeted bisulfite sequencing (TBS) in human buccal swab samples. TBS allows us to measure DNA methylation patterns across reads and enumerate methylation patterns at specific loci. We can then compute the entropy, or distribution, of these patterns. We measured the changes of entropy with age at specific loci and compared these to the age associated changes in the methylation of individual sites. Finally, we tested the ability of these metrics to predict chronological age using penalized regression methods. Our findings demonstrated that entropy-based clocks predict chronological age with similar accuracy as clocks based on the average methylation levels of individual sites, supporting the notion that epigenetic information changes with age.

## RESULTS

### Age associated changes in DNA methylation

To measure age associated changes in DNA methylation, we collected buccal swabs from 100 individuals ranging from 7.2 to 84 years old. The DNA methylation profiles were generated using targeted bisulfite sequencing as described in Methods. Our target panel contained approximately 3000 regions that were selected to cover age associated CpG sites that were identified in multiple epigenetic clocks (Supplementary File 1). Each probe is 120 base pairs, and therefore captures a region of DNA that is slightly larger than the probe length. We obtained an average coverage of 293 reads per sample across these regions. The reads from each sample were aligned to the target loci using BSBolt. The multiple alignments were further refined using the multialign function in MATLAB.

We first calculated the mean methylation of each CpG site in each of the 3000 loci across the 100 samples, and then averaged these levels over a region. We also computed the Cellular Heterogeneity-Adjusted cLonal Methylation (CHALM) [23]. This approach computes the read level methylation of a region after reads are dichotomized into methylated or unmethylated based on the presence of one or more methylcytosines. We also computed the methylation entropy for each locus using four CpG sites within each region, using the Shannon entropy formula. With four CpG sites, there are 16 possible methylation states, and we computed the probability of each state as well as the entropy of the four CpG sites.

**Figure 1:**
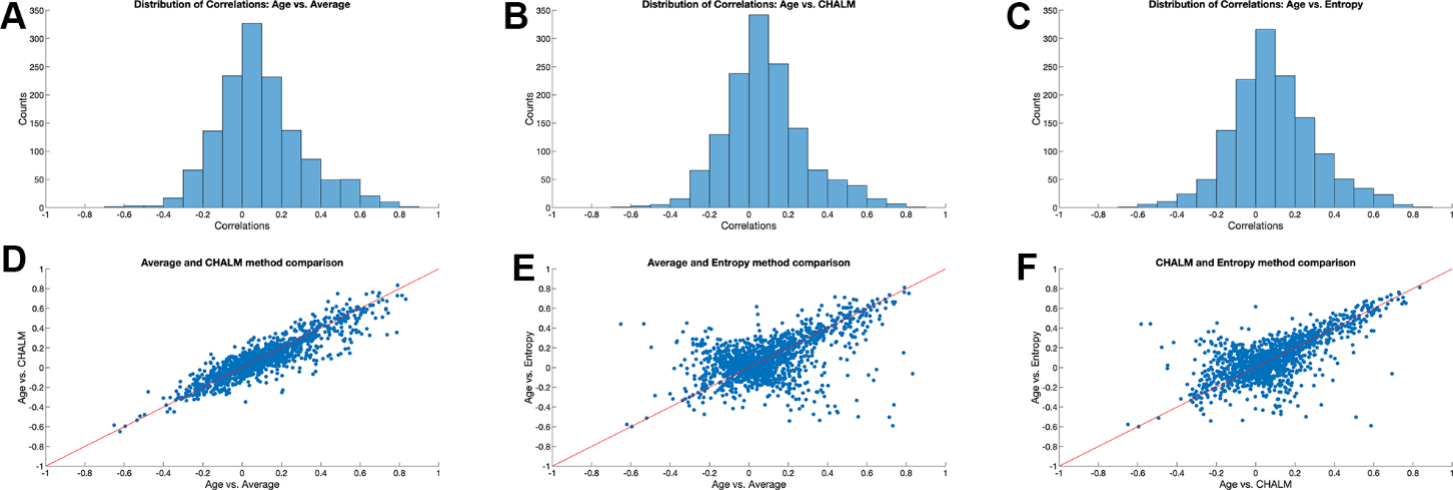
**Correlation between age and three DNA methylation metrics.** (**A**–**C**) Distribution of Pearson’s correlation coefficient between sample age and average methylation, CHALM, and methylation entropy across 3015 loci. (**D**–**F**) Comparison of Pearson’s correlation coefficient for different DNA methylation metrics across 3015 loci.

We next computed the distributions of correlations of each metric with age across the sites and found that they were similar with a bias towards positive correlations and extreme values around -0.6 and 0.8. The correlations of each metric with age followed similar distributions that were approximately normal (Figure 1A–1C).

We next generated scatter plots that compared the values of the three metrics across loci. Age-related changes in mean methylation and CHALM were strongly correlated with a Pearson’s correlation coefficient of r=0.90 (Figure 1D). By contrast, the scatter plots of entropy versus mean methylation or CHALM resulted in more complex patterns with both positive and negative trends (Figure 1E, 1F). The positive diagonal relationship was more prominent, indicating that many loci show increases in entropy and mean methylation with age. Loci in the lower left-hand quadrant appear to become unmethylated with age, with the methylation patterns becoming less disordered. The negative diagonal pattern reveals the presence of loci that lose methylation with age while leading to more random methylation patterns, as well as loci that gain methylation with age, with the methylation patterns becoming more orderly. This demonstrates that methylation entropy is measuring different properties of a locus compared to mean methylation and CHALM, and that loci can become both more or less disordered with age, independently of whether the methylation is increasing or decreasing with age.

### Analysis of specific loci

**Figure 2:**
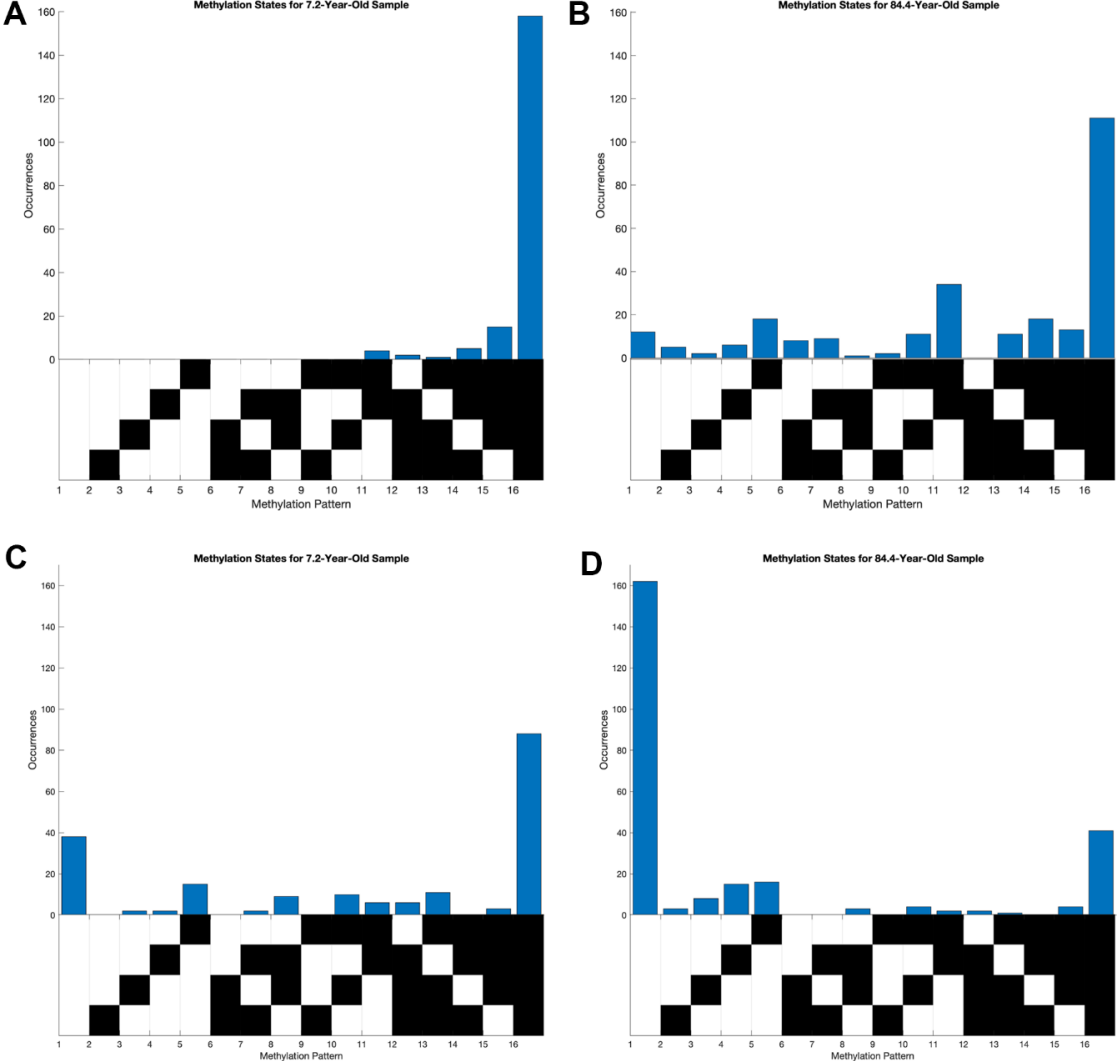
**Distribution of methylation states of young and old samples at loci highly correlated between entropy and age. Each column of the grid below the histograms corresponds to a distinct methylation pattern, where black corresponds to an unmethylated CpG site and white corresponds to a methylated one.** (**A**, **B**) Methylation pattern distributions at chr15:51681883-51681783, where average methylation and methylation entropy were positively correlated with age. (**C**, **D**) Methylation pattern distribution at chr2:101001739-101001859, where average methylation and methylation entropy were positively and negatively correlated with age, respectively.

To better understand the global trends across all loci, we focused our attention on two loci that show extreme correlation of entropy and age. The locus at chr15:51681883-51681783 was of particular interest due to its high positive correlation between entropy and age as well as average methylation and age. The correlation coefficient between age and average methylation was 0.82 and that between age and entropy was 0.79 (Supplementary Figure 1A, 1B). The youngest sample of 7.2 years had mostly hypomethylated reads while the oldest sample of 84.4 years had a much broader occurrence of distinct methylation patterns (Figure 2A, 2B). Though the entirely unmethylated pattern was most prominent in both the young and old individuals, the old sample had a substantial number of reads with partial methylation. We also focused on the locus at chr2:101001739-101001859 because of its prominent negative correlation between entropy and age but high positive correlation between average methylation and age. The correlation coefficient between age and average methylation was 0.73 and that between age and entropy was -0.63 (Supplementary Figure 1C, 1D). As expected, the 7.2-year-old sample had many more occurrences of distinct methylation patterns, while the 84.4-year-old sample primarily had fully methylated patterns (Figure 2C, 2D).

### Epigenetic clocks for predicting age

**Figure 3:**
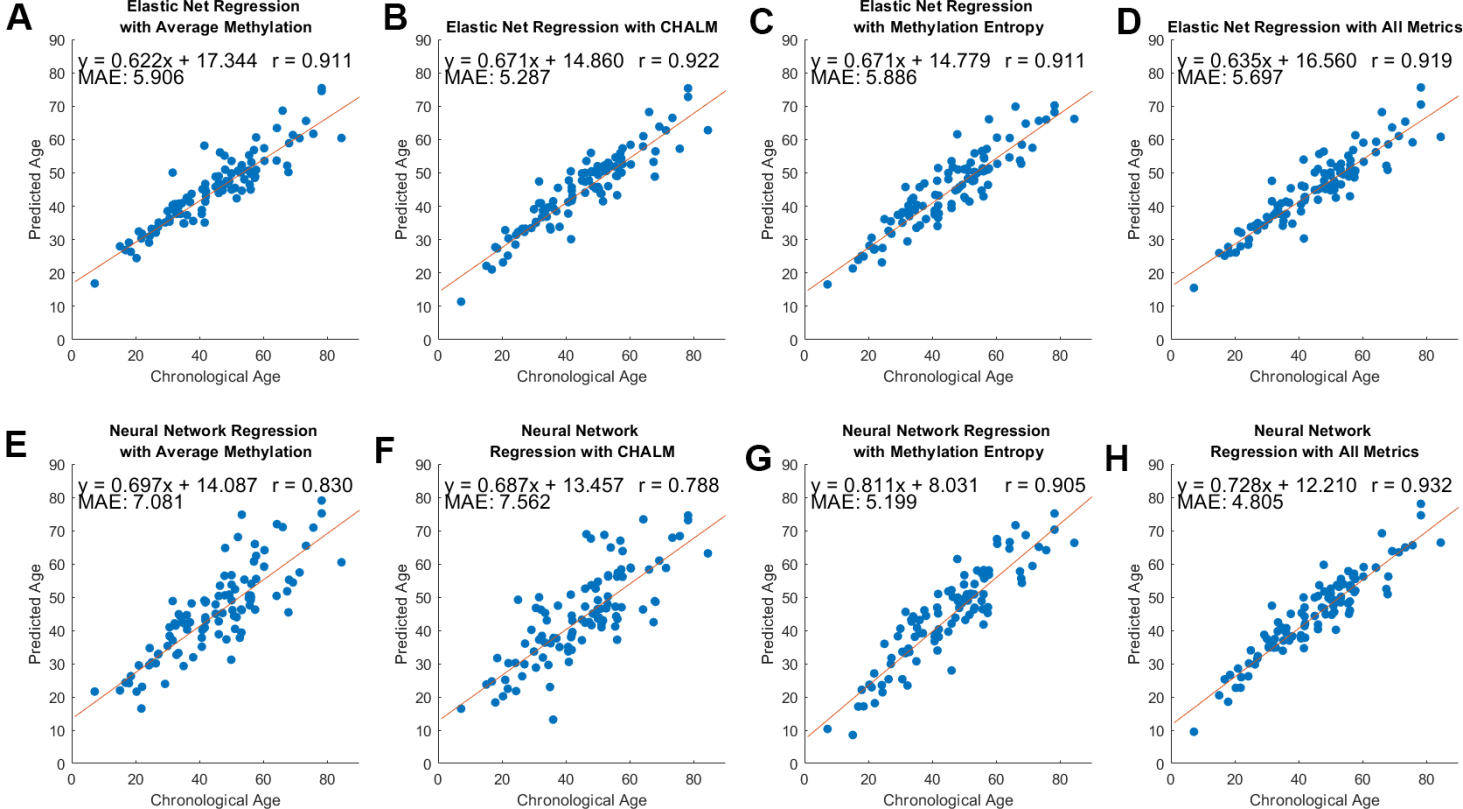
**Model performance across three methylation metrics and two regression methods.** Pearson’s correlation coefficient, equation of best-fit line, and MAE between predicted and chronological age are included. (**A**–**D**) Predicted versus chronological age using average methylation, CHALM, entropy, and a combination of these metrics with elastic net regression. (**E**–**H**) Predicted versus chronological age with neural network regression.

We predicted sample ages using average methylation, CHALM, and entropy separately, then with a combination of those metrics. We utilized elastic net regression and neural network regression as described in Methods. The clocks were evaluated using leave-one-out cross-validation and their performance was measured using the Pearson Correlation between predicted and actual age and the mean absolute error of the predicted age (Figure 3). For the regularization of our neural networks, we used the ‘Lambda’ option which uses Mean Square Error and ridge (L2) regression to prevent overfitting. The structure and activation functions for our neural network models are detailed in Supplementary Table 1.

The Pearson’s correlation coefficient between the predicted and actual ages ranged from 0.79 and 0.93, and the mean average error (MAE) ranged from 4.81 to 7.56 years. Using elastic net regression yielded models that had correlation values consistently over 0.9 and MAE under 6 years for all metrics (Figure 3A–3D). The average number of loci that were contained in the training data after applying elastic net regression were 47, 55, 57, and 56 for the models using average methylation, CHALM, methylation entropy, and all metrics, respectively. By contrast, neural net regression produced models with more variability in performance. Using neural net regression, the mean-based metrics (average methylation and CHALM) had correlation coefficients below 0.9 and MAE greater than 7 years (Figure 3E, 3F). However, the neural net regression models with entropy and all three metrics had correlation coefficients above 0.9 and the lowest MAE among all models (Figure 3G, 3H). The elastic net regression models had a greater tendency than neural network models to overestimate age for young samples and underestimate age for old samples (Supplementary Figure 2). This is unsurprising given the greater flexibility and ability to capture complex relationships of neural network regression compared to elastic net regression. To compare the prediction quality of our clocks to clocks trained on known age-associated CpGs, we repeated the analysis using methylation levels of individual CpG cites from Horvath’s epigenetic clock [12]. The clock was trained on the average methylation levels at the 325 CpG sites that our data shared with Horvath. We applied elastic net regression to this data to predict sample ages. The resultant model had a correlation coefficient of 0.85 and MAE of 7.11 years (Supplementary Figure 3), meaning that its performance was comparable to our two mean methylation-based models (Figure 3A–3D). Together, these results show that with neural network regression, models that utilize methylation entropy can effectively predict chronological age based on epigenetic data. The prediction quality of these models may be similar or slightly better than models that strictly use mean-based methylation metrics. Model performance may be maximized by using a combination of methylation entropy and mean-based methylation metrics.

## DISCUSSION

In this study, we investigated the use of multiple metrics for measuring age-associated changes in DNA methylation. Much of the previous literature relied on the use of DNA microarrays to measure DNA methylation, and since these generate measurements of single nucleotides, only the methylation levels of individual sites have been considered for constructing epigenetic clocks. By contrast, if methylation is measured using bisulfite sequencing, it is possible to measure methylation patterns across CpG sites within the same reads. This property has been leveraged previously to measure read level properties of loci, as implemented in the CHALM metric. Here we also consider Shannon’s entropy to quantify the diversity of methylation states across multiple CpGs at individual sites.

We compared these three metrics in a dataset of 100 individuals of varying ages. The methylation levels were measured across approximately 3000 loci using targeted bisulfite sequencing, allowing us to compute both single cytosine-based metrics as well as read-based metrics. We first computed the distribution of correlations with age for each metric and found that they were broadly similar. However, while the correlations with age for mean methylation and CHALM were positively correlated with each other, age associated correlations with entropy showed more complex patterns. These patterns reveal that the entropy of these loci can both increase or decrease with age, in a manner that is not dependent on whether the methylation of the site increases or decreases with age. This is due to the fact that if early in life a locus is either mostly hypo- or hypermethylated, then with age the diversity of patterns, and hence entropy, will increase. Conversely, if sites start at high entropy early in life, a state typical of hemimethylated regions, then they may lose diversity and entropy with age. Simulations have demonstrated that any normalized biological dataset with accumulating stochasticity is sufficient to predict aging, with an increase in stochastic variation accelerating the aging process and a decrease decelerating it [24].

We next asked whether we could compare the use of these three metrics to construct epigenetic clocks that predict the age of each individual. Selecting only four CpG sites per region to calculate entropy was sufficient to achieve chronological age estimates that were correlated with the actual age above r=0.9. The MAE using neural network regression for entropy was 5.199, which was lower than the other mean-based methods that incorporated many more CpG sites. This suggests that the entropy of a locus is potentially a more useful biomarker of aging than the methylation level of individual sites. Though the 3000 loci analyzed may or may not be representative of the whole genome, this suggests that the entropy of an organism’s methylation profile is informative of its epigenetic age, supporting the Information Theory of Aging [16].

Though entropy and average methylation were similarly correlated with age, an increase in entropy was not strictly associated with an increase in average methylation level. Our analysis revealed that a substantial subset of loci became more methylated with age but decreased in entropy (Figure 1D, 1E), suggesting that these loci converge to a common methylation pattern over time. Future work may identify the mechanisms that lead to the observed relationships between entropy and aging. Furthermore, expanding entropy analysis to different tissues may broaden our understanding of the function of these methylation patterns across cell types.

While we have demonstrated that entropy-based clocks more accurately predict chronological age at this set of loci compared to mean-based quantifications of methylation, these approaches can only be applied to sequence-based methylation measurements. Entropy, which is a fundamentally different measurement of DNA methylation compared to the mean methylation and CHALM, appears to be better equipped to address stochasticity in epigenetics. The normalized methylation entropy (NME) has previously been shown to be associated with higher-order chromatin organization [25] and gene expression variability [26]. Higher NME was associated with lower gene expression levels but higher expression variability in patients with pediatric acute lymphoblastic leukemia [27]. The entropy of an organism’s methylome is therefore an informative metric that appears to be associated with chromatin, transcription, and now aging.

## MATERIALS AND METHODS

### Targeted bisulfite sequencing

DNA was extracted from the buccal swabs using the vendor supplied protocol. The samples were collected as part of a collaboration with Appalachian University. This collaboration led to the collection of multiple types of samples and datasets, some of which have already been published [28, 29]. Buccal swabs were incubated overnight at 50° C before DNA extraction. We applied TBS-seq to characterize the methylomes of the 100 samples. The protocol is described in detail in Morselli et al. [30]. Briefly, 500 ng of extracted DNA were used for TBS-seq library preparation. Fragmented DNA was subject to end repair, dA-tailing and adapter ligation using the NEBNext Ultra II Library prep kit using custom pre-methylated adapters (IDT). Pools of 16 purified libraries were hybridized to the biotinylated probes according to the manufacturer’s protocol. Captured DNA was treated with bisulfite prior to PCR amplification using KAPA HiFi Uracil+(Roche) with the following conditions: 2 min at 98° C; 14 cycles of (98° C for 20 sec; 60° C for 30 sec; 72° C for 30 sec); 72° C for 5 minutes; hold at 4° C. Library QC was performed using the High-Sensitivity D1000 Assay on a 2200 Agilent TapeStation. Pools of 96 libraries were sequenced on a NovaSeq6000 (S1 lane) as paired-end 150 bases [31]. The 3015 probes are available in Supplementary File 1, and the sequencing data are available upon request.

### Data processing

We extracted the sequences corresponding to each of the probes from the Genome Reference Consortium Human Build 38 patch release 14 (GRCh38.p14, or hg38) through bedtools v2.30.0. Reads were aligned to the human genome using BSBolt Align, which generated compressed binary alignment files (BAM) in the SAM format [32]. For each of these loci we extracted the reads from each sample using the samtools view function (samtools 1.14). We then used the multialign algorithm on MATLAB R2023a to align the reads to the template sequence with the GapOpen parameter set to 50 and terminalGapAdjust set to true. We used the subsequent alignment to represent each sample’s methylation profile as a binary matrix. Each row was a different read and each column was a different CpG site. Methylated cytosines were represented in the matrix by 1, whereas unmethylated cytosines were a 0. Occasionally, a read would have an A, G, or null value (if a read did not have coverage over the entire locus) where a cytosine was expected. These entries were expressed as NaN. The general workflow for our data processing is summarized in Supplementary Figure 4.

### Methylation analysis

The traditional method for quantifying methylation levels in a data region is the mean methylation [33], which we computed as


$$\frac{1}{n}\sum_{k}^{n}\;\;\frac{1}{m_{k}}\;\;\sum_{i}^{m_{k}}\;\;c_{ik}$$

where *n* is the number of reads, *m_k_* is the number of CpGs at read *k*, and *C_ik_* is 1 if the *i^th^* CpG site at the *k^th^* read is methylated, and 0 otherwise.

To adjust for cellular heterogeneity, we also calculated CHALM as shown by Xu et al. with the formula


$$\frac{n_{m}}{n_{m}+n_{u}}$$

where *n_m_* and *n_u_* are the counts of methylated and unmethylated reads, respectively [23]. Reads with at least one 5-mC were defined as methylated.

Shannon’s entropy estimates the distribution of methylated states and measures the randomness of information within a given dataset [34]. To compute entropy, we first selected the four most highly covered CpG sites for each locus. This required vertically concatenating the binary matrices across all samples for each locus. We then removed the CpG sites and reads that had NaN in at least half the entries. From this filtered matrix, we extracted the four CpG sites that had the highest coverage (i.e. the least number of NaN entries). The matrix of these four CpG sites was then imputed with the knnimpute command in MATLAB, which applies the k nearest-neighbor method. Since four CpG sites yielded 2^4^ possible methylation patterns, we created a 1x16 vector where each entry corresponded to a distinct methylation pattern, and we computed the number of times each pattern was present in the region. At each locus, only the methylation patterns from samples that had at least 50 reads were included. Finally, the entropy was calculated using the formula


$$-\sum_{i=1}^{16}\;\;p_{i}log\;\;(p_{i})$$

where *p_i_* is entry *i* in the probability vector of methylation patterns. An example of the three computational methods applied is shown in Supplementary Figure 4B.

### Correlation

Each of these metrics were correlated with age for each of the samples using the Pearson correlation coefficient, which was calculated as


$$\rho (A,\;B)=\frac{1}{N-1}\sum_{i=1}^{N}\;\left(\frac{A_{i}-\mu_{A}}{\sigma_{A}}\right)\;\;\left(\frac{B_{i}-\mu_{B}}{\sigma_{B}}\right)$$

where *N* is the number of samples and *A_i_* and *B_i_* are the age and methylation metric of sample *i*, respectively. By substituting sample age for *A_i_* and predicted age for *B_i_*, this method was also used to quantify the performance of each model. Similarly, by substituting the number of loci for *N* and different methylation metrics for *A_i_* and *B_i_*, Pearson’s correlation coefficient was calculated between the three metrics at each locus.

### Modeling age

To predict age based on the three metrics, we used multiple regression techniques. For each locus, we filtered CpG sites with no coverage and imputed the rest of the data using knnimpute. The machine learning models for predicting age were trained using leave-one-out-cross validation (LOOCV). For the first regression technique, we used elastic net regression using the lasso command in MATLAB. The lasso command has a parameter, alpha, that specifies the weight of lasso versus ridge optimization. Various values of alpha were tested, and a value of 0.75, which yielded the best elastic net models, was selected. We used the largest lambda value such that the mean squared error (MSE) was within one standard error of the minimum MSE. For the second regression technique, we used neural network regression using the fitrnet command on MATLAB. We optimized the following hyperparameters using the OptimizeHyperparameters option set to auto: layer sizes, lambda, activation, and standardize. To evaluate the strength of each model, we calculated the goodness-of-fit value and the slope of the best-fit line for each model between the epigenetic and chronological ages. The mean absolute error (MAE) was calculated according to the formula


$$\frac{1}{n}\sum_{i=1}^{n}\;\left|e_{i}\right|$$

where *n* is the number of loci and *e_i_* is the difference between the epigenetic and chronological age at locus *i*.

### Data availability

The code for this project is available at github.com/jonathanchan01/entropy-aging, and the sequencing data are publicly available on the GEO repository at accession number GSE288139.

## Supplementary Material

Supplementary Figures

Supplementary Table 1

Supplementary File 1
