# Supplementary material for: DNA methylation entropy is a biomarker for aging
Source: Aging (Albany NY). 2025 Mar 12;17(3):685–98. doi: 10.18632/aging.206220 (PMC11984425; doi:10.18632/aging.206220)
Supplement: Supplementary Figures [file aging-17-206220-s001.pdf]

SUPPLEMENTARY FIGURES

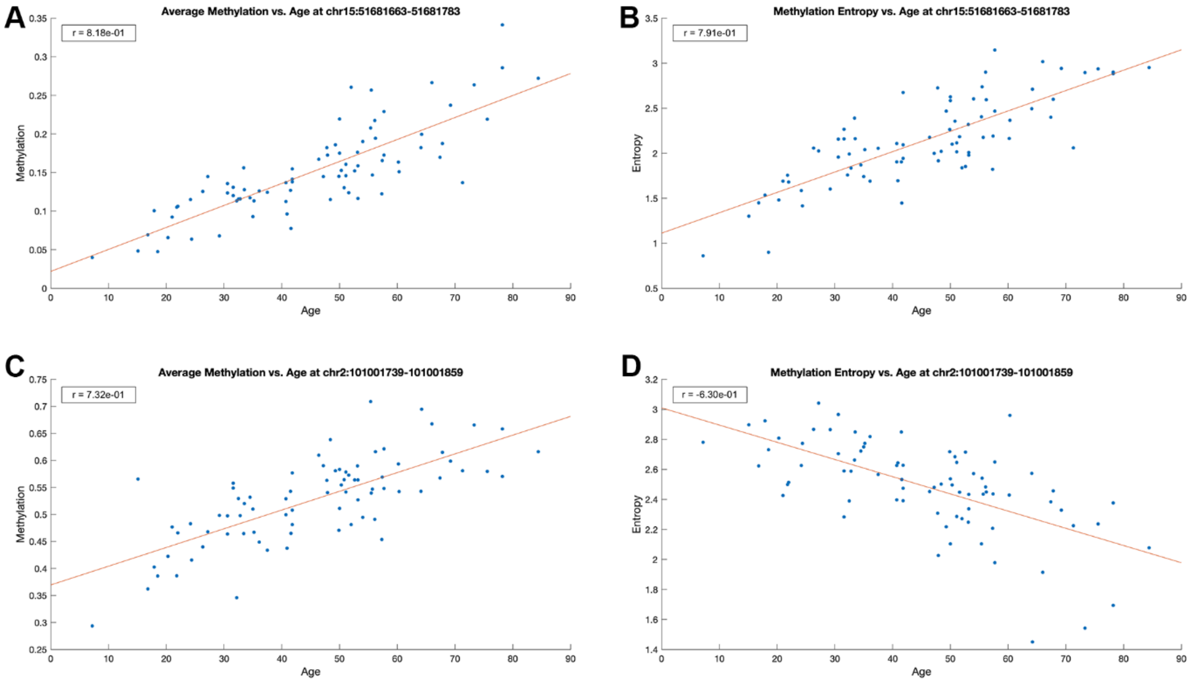

Supplementary Figure 1. Scatterplots comparing age and methylation metrics at specific loci across all samples. (**A**) Average methylation versus age at chr15:51681883-51681783. (**B**) Entropy versus age at chr15:51681883-51681783. (**C**) Average methylation versus age at chr2:101001739-101001859. (**D**) Entropy versus age at chr2:101001739-101001859.

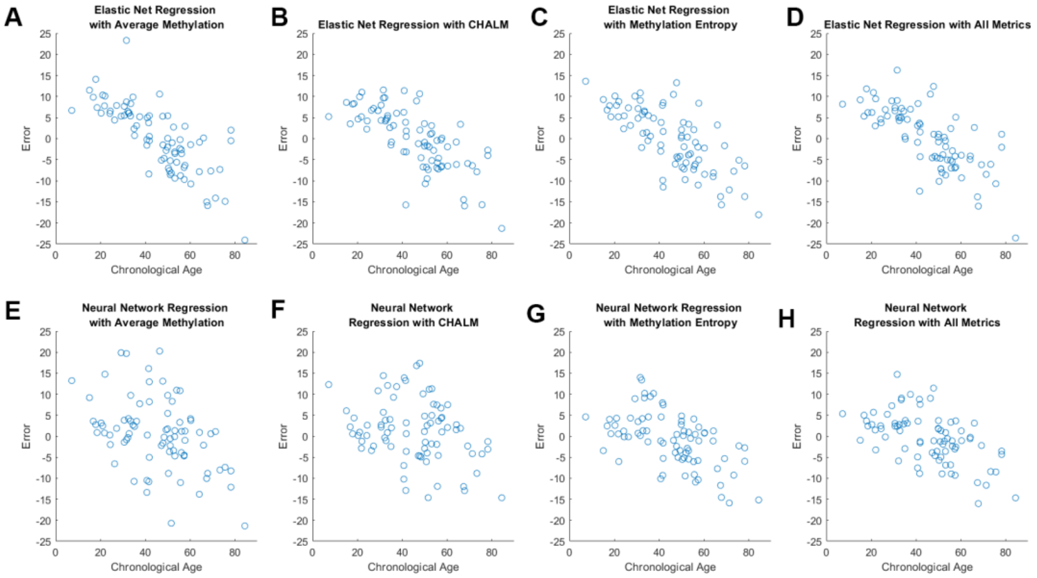

Supplementary Figure 2. Scatterplots comparing the error for each model with actual ages across all samples. Error was calculated as the absolute value of the difference between the predicted age and actual chronological age. (**A**–**D**) Error across samples using average methylation, CHALM, entropy, and all three metrics with models using elastic net regression. (**E**–**H**) Error across samples using average methylation, CHALM, entropy, and all three metrics with models using neural network regression.

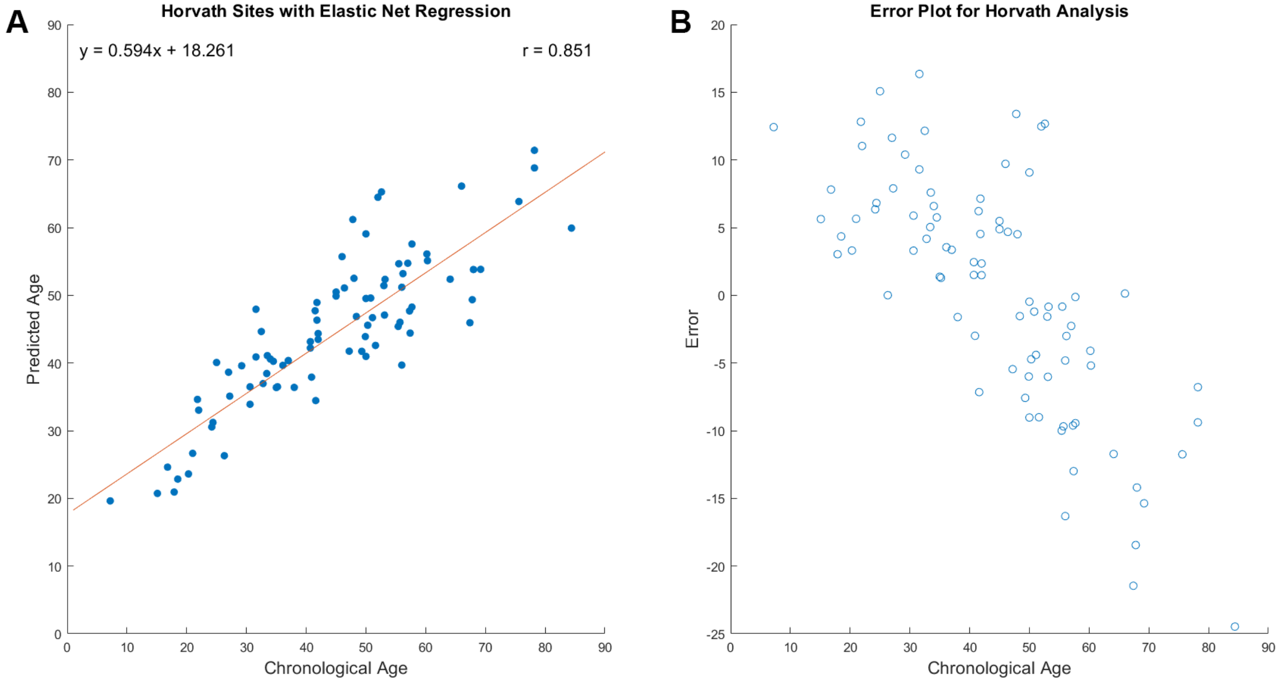

Supplementary Figure 3. Epigenetic clock performance with the 325 CpG sites our data shared with Horvath's study. Error, calculated as the absolute value of the difference between the predicted age and actual chronological age, averaged 7.11 years. (**A**) Predicted versus chronological age using average methylation with elastic net regression. (**B**) Error versus chronological age across all samples.

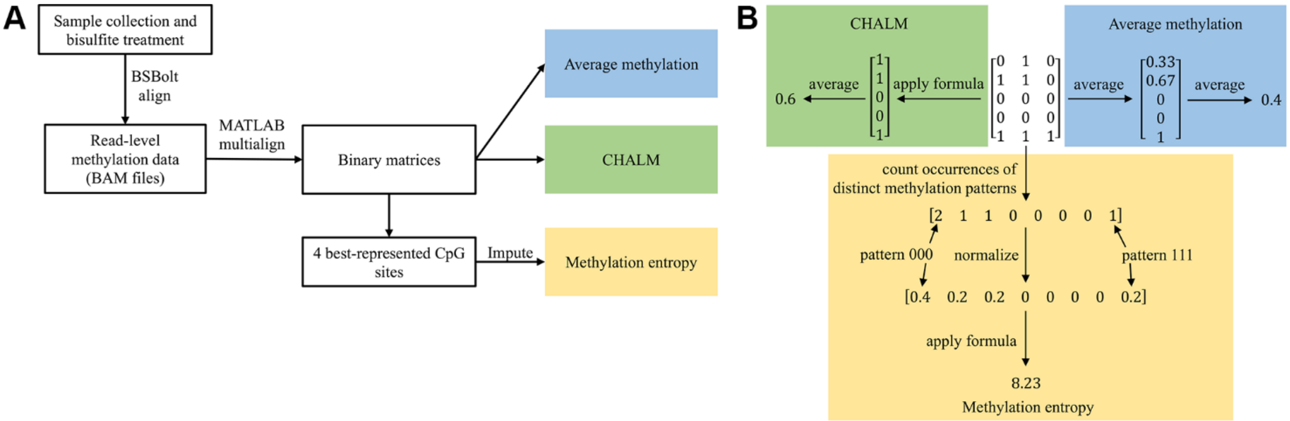

Supplementary Figure 4. Project schematics. (**A**) Flow of data from buccal swab to various DNA methylation metrics. (**B**) Calculation of DNA methylation metrics using an example binary matrix.
