# Supplementary material for: DNA methylation entropy is a biomarker for aging
Source: Aging (Albany NY). 2025 Mar 12;17(3):685–98. doi: 10.18632/aging.206220 (PMC11984425; doi:10.18632/aging.206220)
Supplement: Supplementary Table 1 [file aging-17-206220-s002.pdf]

SUPPLEMENTARY TABLE

Supplementary Table 1. Specifications optimized by setting the OptimizeHyperparameters argument to "auto" in the fitrnet command.

Neural network model specifications				
Metric	Activation	Standardize	Lambda	LayerSizes
Average methylation	Tanh	False	6.0926e-4	296, 11, 10
CHALM	Tanh	False	5.2966e-3	105
Methylation entropy	None	False	4.4047e-3	2, 5
All metrics	None	True	0.46904	162, 293, 15

This minimizes cross-validation loss by using Bayesian optimization. LayerSizes specifies the sizes of each fully connected layer in the neural network model, not including the size of the final fully connected layer.
